# Editorial: Advances in molecular targeted therapies of urologic cancers

**DOI:** 10.3389/fonc.2022.1062015

**Published:** 2022-10-19

**Authors:** Bianca Nitzsche, Michael Höpfner

**Affiliations:** ^1^ Charité – Universitätsmedizin Berlin, Berlin, Germany; ^2^ HumboldtUniversität zu Berlin, Institute for Physiology, Berlin, Germany

**Keywords:** molecular targeted therapy, bladder cancer (BCa), prostate cancer, circRNA, kidney cancer

Urologic cancer is a generic term for distinct malignancies of the urogenital tract comprising of solid tumors of different organs such as bladder, prostate, kidney, urothelium and testicles. Except for testicular cancer rather showing an increased prevalence and incidence in younger adults, most urologic cancers develop in the older male population. All types of urologic cancers represent a problem of major concern and some of them (e.g. prostate cancer) rank among the three most frequent cancers, worldwide and account for a large number of cancer related deaths per year (1). And even more alarming the incidence of urologic cancer is rising due to demographic reasons such as population growth and aging but also due to environmental factors, which have been linked to the development and growth of single urologic cancers, too (e.g. kidney, prostate, bladder) (2).

In the past decade, the understanding of dysregulated pathways in urologic cancers as well as the identification of potential biomarkers helped to identify new targets and pathways for novel therapeutic approaches. In this respect new immunomodulatory substances, antiangiogenic agents, growth factor receptor inhibitors or the targeting of epigenetically modulated signaling pathways have become interesting and promising starting points for future medical treatment of urologic cancer. Besides being more effective, targeted approaches also aim at reducing the severe short- and long-term side effects known for conventional chemo- and radiotherapies.

The idea of the Research Topic “*Advances in molecular targeted therapies of urologic cancers*” was to bring together experts in the field reporting on recent (pre-)clinical findings and achievements and to give an overview on possible therapeutic developments and biomarker analysis in urologic cancer research.

## Prostate cancer

Prostate Cancer (PCa) is the 2^nd^ most diagnosed cancer in men over the last years with more than 1.4 million new cases worldwide in the year 2020 and it is also the 2^nd^ leading cause of cancer death in men for incidence (1, 3). Usually, radical prostatectomy is the main treatment option for localized PCa, and the prognosis is generally favorable. However, in metastatic diseases and in case of a postoperative biochemical recurrence or the development of castration-resistant PCa the outcome for patients remains to be poor (4). The Gleason score is the main criteria for histological staging and for the prediction of outcomes in PCA patients. The main biomarker currently used for the diagnosis of PCa is prostate-specific antigen (PSA) but there is no consensus about the optimal concentration to assess patients prognosis (5). Here novel marker may help to improve the treatment strategy and survival in the affected patients. Circular RNAs (circRNAs) are a novel class of non-coding RNAs (ncRNAs) which have been found to show extensive dysregulation in a handful of human diseases including cancers and their role and their potential has been intensively reviewed and discussed by Taheri et al.. In their review they listed relevant findings about circRNAs that are up- or downregulated and with diagnostic or prognostic values in PCa. Here for circRNA such as circ-ITCH and circMBOAT2, a correlation has been recognized between circRNA expression levels and prognosis in PCa patients. Further studies are needed to determine precisely which circRNA or which set of circRNAs could be used for PCa diagnosis and prognosis.

More targeted treatment options and the improvement of early detection and prognostic values would facilitate reductions in prostate cancer related deaths. Thereby the need to update existing therapy options and invest in the search for novel alternative therapies remains to be a challenge in the future. In line with that, the review “*From Therapy Resistance to Targeted Therapies in Prostate Cancer*” of Moreira-Silva et al. summarized the current state of targeted therapies in PCa and discussed the role and the mechanisms underlying therapy resistance in PCa. They also suggest that selective drug targeting, either alone or in combination with standard treatment options, might improve therapeutic sensitivity of resistant PCa. For castration resistant prostate cancer in non-metastatic or metastatic variants, the therapy options are limited, and further improvements are urgently needed. Taken into account that histone deacetylases are upregulated in many cancer cell types including prostate cancer, molecules that inhibits these epigenetic enzymes have the potential to overcome drug resistance in prostate cancer and promising clinical trials are currently ongoing (6).

## Bladder cancer

Bladder cancer is a common type of cancer that originates in the urinary tract. According to Global Cancer Risk, this cancer caused a total of 570,000 new cases and 200,000 deaths worldwide in 2020 (1). Clinically, 30% of bladder cancer patients present with invasive tumors when initially diagnosed. Even if curative surgery is available, these patients have a 5-year survival rate of only 50%. Therefore, new efforts are needed to investigate the biological causes of bladder cancer progression, and further develop better diagnostic and therapeutic modalities. The study by Dong et al. explored the role of the transmembrane glycoprotein receptor neuropilin.1 (NRP1) in bladder cancer and provided evidence for specific NRP1 expression patterns and could show that inhibiting NRP1 expression could promote apoptosis and suppress proliferation, angiogenesis, migration, and invasion of BC cells, implying the NRP1 as an potential target in BD therapy. In the last years various novel antitumor treatments have been developed for various cancer entities as well as bladder cancer and immunotherapeutic approaches are among the most promising. Immunotherapy aims to activate the immune system to target uncontrolled cancer cell proliferation within the body. Accordingly, two articles in the present Research Topic deal with the immune characteristics of bladder cancer cells as well as the evaluation of important side effects of immunotherapy for bladder cancer. Both adding new and intriguing aspects and information on this emerging topic (Zhu et al.; Lou et al.).

In conclusion, several pathways have been explored as potential targets for enhanced urologic cancer treatment in the future. The present Research Topic aimed adding new aspects to the developmental status of targeted therapies, especially in prostate and bladder cancer and brought forth a Research Topic of intriguing studies on the suitability of novel single agents or combination therapies to fight urologic cancers.

## Author contributions

Both authors have made a substantial, direct, and intellectual contribution to the work and approved it for publication.

## Conflict of interest

The authors declare that the research was conducted in the absence of any commercial or financial relationships that could be construed as a potential conflict of interest.

## Publisher’s note

All claims expressed in this article are solely those of the authors and do not necessarily represent those of their affiliated organizations, or those of the publisher, the editors and the reviewers. Any product that may be evaluated in this article, or claim that may be made by its manufacturer, is not guaranteed or endorsed by the publisher.

## References

[1] Sung H Ferlay J Siegel RL Laversanne M Soerjomataram I Jemal A. Global cancer statistics 2020: GLOBOCAN estimates of incidence and mortality worldwide for 36 cancers in 185 countries. CA Cancer J Clin (2021) 71:209–49. doi: 10.3322/caac.21660 33538338

[2] Dy GW Gore JL Forouzanfar MH Naghavi M Fitzmaurice C. Global burden of urologic cancers, 1990–2013. Eur Urol (2017) 71:437–46. doi: 10.1016/j.eururo.2016.10.008 28029399

[3] Siegel RL Miller KD Fuchs HE Jemal A. Cancer statistics, 2022. CA Cancer J Clin (2022) 72:7–33. doi: 10.3322/caac.21708 35020204

[4] Hull GW Rabbani F Abbas F Wheeler TM Kattan MW Scardino PT. Cancer control with radical prostatectomy alone in 1,000 consecutive patients. J Urol (2002) 167:528–34. doi: 10.1016/S0022-5347(01)69079-7 11792912

[5] Asso RN Degrande FAM Fernandes da Silva JL Leite ETT. Postoperative radiotherapy in prostate cancer: When and how? – an update review. Cancer (2022) 26:742–8. doi: 10.1016/j.canrad.2021.10.009 35428564

[6] Biersack B Nitzsche B Höpfner M. HDAC inhibitors with potential to overcome drug resistance in castration-resistant prostate cancer. Cancer Drug Resist Alhambra Calif (2022) 5:64–79. doi: 10.20517/cdr.2021.105 PMC8992583 35582529

